# Off-Grid DOA Estimation Using Sparse Bayesian Learning for MIMO Radar under Impulsive Noise

**DOI:** 10.3390/s22166268

**Published:** 2022-08-20

**Authors:** Jitong Ma, Jiacheng Zhang, Zhengyan Yang, Tianshuang Qiu

**Affiliations:** 1 College of Information Science and Technology, Dalian Maritime University, Dalian 116026, China; 2 School of Artificial Intelligence, Nanjing University of Information Science and Technology, Nanjing 210044, China; 3 Department of Electronic Information and Electrical Engineering, Dalian University of Technology, Dalian 116024, China; 4 College of Transportation Engineering, Dalian Maritime University, Dalian 116026, China

**Keywords:** DOA estimation, monostatic MIMO radar, impulsive noise, sparse Bayesian learning

## Abstract

Direction of arrival (DOA) estimation is an essential and fundamental part of array signal processing, which has been widely used in radio monitoring, autonomous driving of vehicles, intelligent navigation, etc. However, it remains a challenge to accurately estimate DOA for multiple-input multiple-output (MIMO) radar in impulsive noise environments. To address this problem, an off-grid DOA estimation method for monostatic MIMO radar is proposed to deal with non-circular signals under impulsive noise. In the proposed method, firstly, based on the property of non-circular signal and array structure, a virtual array output was built and a real-valued sparse representation for the signal model was constructed. Then, an off-grid sparse Bayesian learning (SBL) framework is proposed and further applied to the virtual array to construct novel off-grid sparse model. Finally, off-grid DOA estimation was realized through the solution of the sparse reconstruction with high accuracy even in impulsive noise. Numerous simulations were performed to compare the algorithm with existing methods. Simulation results verify that the proposed off-grid DOA method enables evident performance improvement in terms of accuracy and robustness compared with other works on impulsive noise.

## 1. Introduction

With the rapid development of wireless communications, such as fifth-generation (5G) communication systems and the upcoming 6G communication systems, various smart technologies have been extended to provide intelligent and effective services to users [1,2,3]. Among these smart technologies, multiple-input multiple-output (MIMO) radar has been extensively applied in many military and civilian fields, such as target detection, intelligent radio monitoring and parameter estimation [4,5,6]. Generally, an MIMO radar system can be divided into two categories: bistatic MIMO radar system and monostatic MIMO radar system. Compared with bistatic MIMO radar, monostatic MIMO radar has larger array aperture and degree of freedom as it is equipped with collocated of phased arrays [4]. Hence, we mainly consider the monostatic MIMO radar system in this paper.

Direction of arrival (DOA) estimation is a fundamental and essential step in target parameter estimation, and it has gained widespread attention in wireless communication and vehicular navigation fields [7]. As MIMO radar can achieve a higher spatial resolution when compared to conventional uniform linear array (ULA), solving DOA estimation with MIMO radar has been a hot topic, especially for monostatic MIMO radar [1,8,9]. Similar to DOA estimation in ULA, subspace based methods are dominated by DOA estimation with MIMO radar, mainly including MUSIC [10], ESPRIT [11], and the parallel factor based method [12]. Nevertheless, these subspace based methods usually suffer performance degradation with less prior knowledge and fewer signal snapshots. Considering these limitations, a promising technique for DOA estimation is further exploited based on sparse representation [13]. Meanwhile, a series of sparse representation-based approaches have been proposed to solve DOA estimation with monostatic MIMO radar. In [14], a reweighted $\ell_{1}$-SVD is utilized for sparse reconstruction to improve the estimation performance. In [15], an improved $l_{p}$ norm minimization DOA estimation method is proposed to separate two closely spaced sources. Ref. [16] proposes a novel idea of simultaneous source number identification and DOA estimation with high accuracy. Ref. [17] further improved the performance for DOA estimation with a non-circular signal property and nuclear norm minimization. In [18], the iterative reweighted proximal projection is firstly introduced to estimate DOA for MIMO radar with effective performance improvement. Ref. [19] proposed a Bayesian learning based DOA approach which also shows resistance to mutual coupling. Non-circular signals can extend the array aperture with a non-circular signal property [20]. The non-circular signal property is employed to estimate DOA with MIMO radar in [21]. Based on this property, both subspace and sparse representation based methods can improve the angular resolution and estimation accuracy.

Although the aforementioned methods have advantages in MIMO radar DOA estimation, their performance would degrade severely in impulsive noise. This is because the background noise is assumed to be AWGN (additive white Gaussian noise) in these DOA estimation methods. However, it is quite unrealistic to adopt this AWGN assumption to describe the noise in complicated electromagnetic environments. Due to the impact of natural or man-made signal sources, the channel noise usually behaves with non-Gaussian impulsive characteristics [22,23]. Under an impulsive noise environment, the conventional DOA estimation methods, which are based on second-order statistics (SOS) or higher-order statistics (HOS), suffer serious performance degradation as SOS and HOS are not convergent for non-Gaussian impulsive noise. To suppress impulsive noise for DOA estimation, many concepts have been developed such as fractional lower-order statistics [24,25,26], correntropy [27,28], nonlinear functions [29,30] and so on. However, there are few works that focus on solving DOA estimation with MIMO radar based on sparse representation in impulsive noise. Robust and accurate MIMO radar DOA estimation in impulsive noise remains a challenge.

To accurately estimate DOA with MIMO radar in impulsive noise, a novel off-grid DOA estimation method is proposed in this paper. In the proposed method, firstly, a real-valued array model is constructed with the aid of the structure of monostatic MIMO radar and non-circular signals’ property. The constructed real-valued virtual array can effectively reduce the computational complexity for sparse Bayesian learning and extend the array aperture. Then, an off-grid sparse Bayesian learning is proposed and applied to the virtual array to solve DOA estimation. Based on the proposed Bayesian learning framework, off-grid DOA can be estimated and meanwhile the impulsive noise can be effectively suppressed. Finally, comprehensive simulations are performed to verify the superiority of the proposed DOA estimation method. Furthermore, the advantages of the proposed method can be given as follows:

1.  The proposed DOA method could accurately estimate DOA for MIMO radar under non-Gaussian impulsive noise without the prior knowledge of its parameters, such as the characteristic exponent of alpha stable distribution. Hence, the proposed method is with wider application scope than conventional DOA methods;
2.  The proposed DOA method can estimate DOA more accurately than the conventional DOA methods, including the methods in Refs. [17,18,31], as an off-grid mechanism is adopted to design the proposed DOA method;
3.  The proposed DOA method has a more robust performance than the conventional DOA estimation methods under impulsive noise, especially in the low generalized signal to noise ratio condition.

The framework of this paper is organized as follows: Section 2 describes the problem formulation. Section 3 explains the derivation of our method. Numerical experiments are presented in Section 4 and conclusions are drawn in Section 5.

Notation: The operator diag{a} denotes a diagonal matrix using the elements in a; ⊙ is the Khatri–Rao product; The operator Tr(A) calculates the trace of A; Matrices $0_{M}$ and $I_{M}$ denote the M×M matrix of zeros and identity, respectively.

## 2. Problem Formulation

### 2.1. Signal Model

Considering a monostatic MIMO radar with *M* transmitted and *N* received sensors, both of the transmitted and received arrays are assumed to be ULA with a half-wavelength space between the adjacent sensors. *K* far-field narrow band signals are assumed to be the signal of interest. Since the located arrays are close in monostatic MIMO radar, the directions of targets on the transmitted and received arrays are assumed to be same with $\theta_{i}\left(i=1,2,\ldots ,K\right)$. Therefore, the output of the array with MN×1 sensors can be expressed as:

(1) x(t)=As(t)+n(t),

where $x\left(t\right)={\left[x_{1}\left(t\right),x_{2}\left(t\right),\ldots ,x_{MN}\left(t\right)\right]}^{T}$, $n\left(t\right)\in C^{MN\times 1}$ denotes the impulsive noise, $s\left(t\right)\in C^{K\times 1}$ is the source signal, A is the steering matrix whose dimension is MN×K, and $A=A_{t}⊙A_{r}$ with $A_{t}=\left[a_{t}\left(\theta_{1}\right),a_{t}\left(\theta_{2}\right),\ldots ,a_{t}\left(\theta_{K}\right)\right]$, $A_{r}=\left[a_{r}\left(\theta_{1}\right),a_{r}\left(\theta_{2}\right),\ldots ,a_{r}\left(\theta_{K}\right)\right]$ and

(2) $$a_{t}\left(\theta_{i}\right)={\left[1,e^{j\pi \sin(\theta_{i})},\ldots ,e^{j\pi \left(M-1\right)\sin\left(\theta_{i}\right)}\right]}^{T},$$

(3) $$a_{r}\left(\theta_{i}\right)={\left[1,e^{j\pi \sin(\theta_{i})},\ldots ,e^{j\pi \left(N-1\right)\sin\left(\theta_{i}\right)}\right]}^{T},$$

where the operator ⊙ is the Khatri–Rao product, and for matrices $F_{p\times n}$ and $G_{q\times n}$, their Khatri–Rao product is defined as $F⊙G=\left[f_{1}⊗g_{1},f_{2}⊗g_{2},\cdot \cdot \cdot ,f_{n}⊗g_{n}\right]\in R^{pq\times n}$ where ⊗ denotes the Kronecker product which is a kind of common operation in matrix analysis.

Collecting *L* snapshots of the received signals, the output can be represented as:

(4) X=AS+N,

where

(5) X=[x(1),x(2),…,x(L)],

(6) S=[s(1),s(2),…,s(L)],

(7) N=[n(1),n(2),…,n(L)].

For a non-circular signal, it can be represented by the real-valued signal $s_{0}$ and the phase Φ, given as:

(8) $$s=\Phi s_{0},$$

(9) $$s_{0}={\left[s_{01},\ldots ,s_{0K}\right]}^{T},$$

(10) $$\Phi =\mathrm{diag}\{e^{j\varphi_{1}/2},e^{j\varphi_{2}/2},\ldots ,e^{j\varphi_{K}/2}\},$$

where Φ is the non-circular phase of the input signal.

### 2.2. Impulsive Noise

In practical wireless communication environments, such as the impact of natural or man-made interference sources, Gaussian distribution is usually not suitable to describe the wireless channel noise. Correspondingly, alpha-stable distribution is usually adopted to describe the features of non-Gaussian impulsive noise [3,27]. As there is no closed probability density function of alpha-stable distribution, the characteristic function is introduced to evaluate the impulsiveness of noise as:

(11) $$\varphi \left(t\right)\;=\;\exp\left\{j\mu t-\gamma {\left|t\right|}^{\alpha }\left[1-j\beta \mathrm{sgn}\left(t\right)\varpi \left(t,\alpha \right)\right]\right\},$$

where

(12) ϖt,α=tanπαπα22α≠122ππlogtα=1,

(13) $$\;\mathrm{sgn}\left(t\right)=\left\{\begin{array}{c} 1,\;t>0\\ 0,\;t=0\\ -1,\;t<0. \end{array}\right.,$$

α∈(0,2] denotes the characteristic exponent, and it mainly controls the thickness of distribution tails. The impulsive noise will produce more outliers with smaller α. γ represents the dispersion parameter with value ranges of γ>0. μ represents the location parameter. β stands for the symmetry parameter taking values −1<β≤1. When β=0, this distribution would become a symmetric alpha stable (SαS) distribution.

In addition, when α<2, the alpha stable distribution exhibits heavier tails and this distribution no longer has the finite SOS or HOS. Hence, the performance of conventional DOA estimation methods which are designed on the basis of SOS or HOS degrade seriously in impulsive noise.

### 2.3. Sparse Representation for MIMO DOA Estimation

Commonly, the dimension reduction is employed in MIMO array output to remove redundant information [11]. The transformation matrix is used to reduce the dimension, given as:

(14) $$U=F^{-1}H^{H},$$

where

(15) $$F=diag(\left[1,2,\ldots ,\underset{|M-N|+1}{\underset{︸}{\min(M,N),\ldots ,\min(M,N)}},\ldots ,2,1\right]),$$

(16) $$H={\left[h_{0},h_{1},,\ldots ,h_{M}\right]}^{T},$$

and the m−th matrix $h_{m}$ in H is defined as:

(17) $$h_{m}={\left[0_{N\times m},I_{N\times N},0_{N\times \left(M-m-1\right)}\right]}^{T},$$

where $I_{N\times N}$ is the unit matrix with the dimension of N×N, and $0_{N\times m}$ and $0_{N\times (M-m-1)}$ denote the zero matrix with the dimension of N×m and N×(M−m−1), respectively.

Multiplying the output X with the transformation matrix U, a virtual array output with dimension reduction can be obtained by:

(18) $$\tilde{X}=UX=F^{1/2}BS+UN=\bar{B}S+\bar{N},$$

where $\tilde{X}\in C^{(M+N-1)\times L}$, $\bar{N}=UN\in C^{(M+N-1)\times L}$, $\bar{B}=F^{1/2}B\in C^{(M+N-1)\times K}$, $B=[b\left(\theta_{1}\right),b\left(\theta_{2}\right),\ldots ,b\left(\theta_{K}\right)]$, $F^{1/2}$ denotes the square root of F and

(19) $$b\left(\theta_{i}\right)={\left[1,e^{j\pi \sin(\theta_{i})},\ldots ,e^{j\pi \left(M+N-2\right)\sin\left(\theta_{i}\right)}\right]}^{T}.$$

Then, apply sparse representation method to $\tilde{X}$, given as:

(20) $$\tilde{X}=\bar{B}\left(\Theta \right)S_{v}+\bar{N},$$

where $\bar{B}\left(\Theta \right)=F^{1/2}B\left(\Theta \right)=F^{1/2}\left[b\left(\theta_{1}\right),b\left(\theta_{2}\right),\ldots ,b\left(\theta_{P}\right)\right]$ and ${\left\{\theta_{i}\right\}}_{i=1}^{P},P\gg K$ denote all possible DOAs in the sampling grid, $S_{v}\in C^{P\times L}$ denotes the sparse representation of S. Only when $\theta_{i}$ equals to the true DOA of received signal, the *i*th row of $S_{v}$ has a non-zero value. Therefore, DOA estimation is transformed to a sparse representation problem and DOA can be estimated by finding the non-zero elements in $S_{v}$ after sparse reconstruction. Moreover, there are also some methods which construct the sparse representation model by exploiting the covariance $R=E[\tilde{X}{\tilde{X}}^{H}]$ based vector. However, the above two models still have some limitations. Specifically, the sparse model of the original output may lead to large computation. The covariance-based model is not useful for coherent or correlated signals and it is also sensitive to the snapshots of signals.

## 3. Proposed Off-Grid DOA Estimation Method

In this section, we propose a novel off-grid DOA estimation method using sparse Bayesian learning for MIMO radar in impulsive noise. The details are presented as follows.

### 3.1. Sparse Signal Model

To construct a model with low computational complexity and meanwhile ensure the model is also suitable for coherent signals, a real-valued transformation is firstly utilized as:

(21) $$X_{R}=\frac{1}{2}\left(\tilde{X}+{\tilde{X}}^{*}\right),$$

(22) $$X_{I}=\frac{1}{2j}\left(\tilde{X}-{\tilde{X}}^{*}\right).$$

Based on the non-circular signals property, Equation (21) and Equation (22) can be expressed as:

(23) $$X_{R}=\frac{1}{2}\left(\bar{B}\Phi +{\bar{B}}^{*}\Phi^{*}\right)S_{0}+Re\left[\bar{N}\right],$$

(24) $$X_{I}=\frac{1}{2j}\left(\bar{B}\Phi -{\bar{B}}^{*}\Phi^{*}\right)S_{0}+Im\left[\bar{N}\right],$$

where the operators Re[·] and Im[·] extract the real and imaginary part of the variable, respectively. After that, we can derive a extended output model as:

(25) $$\bar{X}=\left[\begin{array}{c} X_{R}\\ X_{I} \end{array}\right]=A_{e}S_{0}+\left[\begin{array}{c} Re[N]\\ Im[N] \end{array}\right],$$

where $A_{e}$ denotes the new steering matrix with

(26) $$A_{e}=\left[a_{e}\left(\theta_{1}\right),\ldots ,a_{e}\left(\theta_{K}\right)\right],$$

(27) $$a_{e}\left(\theta_{i}\right)=\left[\begin{array}{c} F_{1}a_{R}\left(\theta_{i}\right)\\ F_{1}a_{I}\left(\theta_{i}\right) \end{array}\right],$$

(28) $$a_{R}\left(\theta_{k}\right)={\left[1,\cos\left(\tau_{k}\right),\ldots ,\cos\left(\left(M+N-1\right)\tau_{k}\right)\right]}^{T},$$

(29) $$a_{I}\left(\theta_{k}\right)={\left[0,\sin\left(\tau_{k}\right),\ldots ,\sin\left(\left(M+N-1\right)\tau_{k}\right)\right]}^{T},$$

where $\tau_{k}=\pi \sin\left(\theta_{k}\right)$, $F_{1}=F^{1/2}$. When the received signals are interfered by impulsive noise, the output of signals can be further denoted by:

(30) $$\bar{X}=A_{e}S_{0}+Z+W=\left[A_{e}\;I_{2Q}\right]\left[\begin{array}{c} S_{0}\\ Z \end{array}\right]+W,$$

where Q=M+N−1, Z represents a matrix which consists of outliers and W is a Gaussian distributed noise. Let $\bar{A}=\left[A_{e}\;I_{2Q}\right]$ and $\bar{S}={\left[S_{0}\;Z\right]}^{T}$, the output can be further represented as:

(31) $$\bar{X}=\bar{A}\bar{S}+W.$$

To construct a sparse model of Equation (31), we firstly consider the off-grid effect. Given a fixed sampling grid $\tilde{\theta }=\left[{\tilde{\theta }}_{1},\ldots ,{\tilde{\theta }}_{P}\right]$ from [−0.5π,0.5π], P≫K. Assuming ${\tilde{\theta }}_{nk}$ is the nearest grid point to the true DOA $\theta_{k}$, then the steering vector of $a_{e}\left(\theta_{k}\right)$ can be approximated by:

(32) $$a_{e}\left(\theta_{k}\right)\approx a_{e}\left({\tilde{\theta }}_{nk}\right)+b_{e}\left({\tilde{\theta }}_{nk}\right)\left(\theta_{k}-{\tilde{\theta }}_{nk}\right),$$

where $b_{e}\left({\tilde{\theta }}_{nk}\right)=a_{e}^{{}^{\prime }}\left({\tilde{\theta }}_{nk}\right)$. Furthermore, the off-grid sparse model of Equation (31) can be expressed as:

(33) $$\bar{X}={\bar{A}}_{v}{\bar{S}}_{v}+W,$$

where ${\bar{S}}_{v}=\left[S_{1},Z\right]$ and $S_{1}$ is the sparse representation of $S_{0}$, ${\bar{A}}_{v}=\left[\bar{A}\left(\Theta \right),I_{2Q}\right]+B_{1}\mathrm{diag}\left(\beta_{1}\right)$, $\bar{A}\left(\Theta \right)=\left[a_{e}\left(\theta_{1}\right),\ldots ,a_{e}\left(\theta_{P}\right)\right]$, $B_{1}=\left[B_{v},0_{2Q}\right]$, $\beta_{1}=\left[\beta_{1},\beta_{2},\ldots ,\beta_{P},0_{1\times 2Q}\right]$ and

(34) $$B_{v}=\left[b_{e}\left({\tilde{\theta }}_{1}\right),\ldots ,b_{e}\left({\tilde{\theta }}_{P}\right)\right].$$

Although the array dimension has increased in this model, real-valued transformation can effectively reduce the computational complexity. This model is also suitable for coherent and correlated signals with high efficiency.

### 3.2. Sparse Bayesian Learning Based Approach

To apply Bayesian inference, we further analyze the distribution of different variables. For the Gaussian distributed noise W, the distribution is assumed as:

(35) $$p\left(W|\varepsilon \right)=\prod_{l=0}^{L-1}\mathrm{CN}\left(W\left(l\right)|0_{2Q\times 1},\varepsilon^{-1}I_{2Q}\right),$$

where W(l) is the *l*th column of W, $\varepsilon ≃\sigma^{-2}$ and $\sigma^{2}$ represents the variance of noise. ε is a hyper prior parameter which is assumed with Gamma distribution,

(36) p(ε)=Γ(ε|1,a).

Then, the distribution of the output can be obtained by:

(37) $$p\left(\bar{X}|{\bar{S}}_{v},\varepsilon ,\beta_{1}\right)=\prod_{l=0}^{L-1}\mathrm{CN}\left({\bar{X}}_{l}|{\bar{A}}_{v}{\bar{S}}_{v}\left(l\right),\varepsilon^{-1}I_{2Q}\right).$$

Since $S_{1}$ is the sparse representation of $S_{0}$, it can be assumed with Gaussian distribution as:

(38) $$p\left(S_{1}|\delta \right)=\prod_{l=0}^{L-1}\mathrm{CN}\left(S_{1}\left(l\right)|0_{P\times 1},\mathrm{diag}{\left(\delta \right)}^{-1}\right),$$

where $\delta =[\delta_{1},\delta_{2},\ldots ,\delta_{P}]$ and $\delta_{i}$ is the variance’s inversion of *i*th row in $S_{1}$. Because δ is still a hyper parameter, Gamma distribution is also considered for δ

(39) $$p\left(\delta \right)=\prod_{n=1}^{P}\Gamma \left(\delta_{n}|1,\xi \right),$$

where ξ is a small value near to zero. Then, an IID (Independent and identically distributed) Gaussian distribution is utilized to model the outlier matrix Z as:

(40) $$p\left(Z|\Lambda \right)=\prod_{i=0}^{2Q-1}\prod_{l=0}^{L-1}\mathrm{CN}\left(Z_{i,l}|0,\lambda_{i,l}^{-1}\right),$$

where $Z_{i,l}$ and $\lambda_{i,l}$ are the (i,l)th element of Z and Λ. Λ is a variance matrix to model the outlier matrix. Gamma distribution is also applied to model the hyper prior of Λ as:

(41) $$p\left(\Lambda \right)=\prod_{i=0}^{2Q-1}\prod_{l=0}^{L-1}\Gamma \left(\lambda_{i,l}|1,\xi \right).$$

As Z and $S_{1}$ are considered independent, the distribution of ${\bar{S}}_{v}$ could be calculated by:

(42) $$p\left({\bar{S}}_{v}|\Omega \right)=\prod_{l=0}^{L-1}\mathrm{CN}\left({\bar{S}}_{v}\left(l\right)|0_{(N_{0}+2Q)\times 1},\Omega_{l}^{-1}\right),$$

where $\Omega_{l}=\mathrm{diag}\left(\left[\delta ,\Lambda_{l}\right]\right)$. Furthermore, the distribution of $\beta_{1}$ is modeled by uniform distribution as:

(43) $$\beta_{1}∼U\left([-0.5r,0.5r]\right),$$

where *r* is the sampling interval of the original coarse grid.

On the basis of these distributions, we can realize sparse reconstruction via maximizing following posterior probability:

(44) $$S_{out}=\arg\max_{{\bar{S}}_{v}}p\left({\bar{S}}_{v}|\bar{X},\varepsilon ,\delta ,\Lambda ,\beta_{1}\right).$$

Based on the above distributions, we can get:

(45) $$p\left({\bar{S}}_{v}|\bar{X},\varepsilon ,\delta ,\Lambda ,\beta_{1}\right)≜\prod_{l=0}^{L-1}\mathrm{CN}\left({\bar{S}}_{v}\left(l\right)|\mu_{l},\Sigma_{l}\right),$$

where

(46) $$\mu_{l}=\varepsilon \Sigma_{l}{\bar{A}}_{v}^{T}\bar{X}\left(l\right),$$

(47) $$\Sigma_{l}={\left(\varepsilon {\bar{A}}_{v}^{T}{\bar{A}}_{v}+\Omega_{l}\right)}^{-1}.$$

With the aid of Equations (46) and (47), sparse reconstruction can be realized. However, Equations (46) and (47) cannot be directly calculated without estimating the unknown parameters. To solve this problem, expectation maximization (EM) approach is introduced to calculate the unknown parameters. Considering the expectation likelihood function with respect to the posterior of ${\bar{S}}_{v}$,

(48) $$\begin{array}{cc} L(\varepsilon ,\delta ,\Lambda , & \beta_{1})=E\left[\ln p\left(\bar{X},{\bar{S}}_{v},\varepsilon ,\delta ,\Lambda ,\beta_{1}\right)\right]\\ \; & =E[\ln p\left(\bar{X}|{\bar{S}}_{v},\varepsilon \right)p\left({\bar{S}}_{v}|\delta ,\Lambda \right)p\left(\varepsilon \right)p\left(\delta \right)p\left(\Lambda \right)p\left(\beta_{1}\right)], \end{array}$$

where E denotes an expectation to the posterior of ${\bar{S}}_{v}$. On the basis of this likelihood function, the hyper prior parameters can be further calculated as follows.

For ε, ignoring the independent terms, the following can be obtained:

(49) $$\begin{array}{cc} \; & L\left(\varepsilon \right)\propto E[\ln p\left(\bar{X}|{\bar{S}}_{v},\varepsilon ,\delta ,\Lambda ,\beta_{1}\right)p\left(\varepsilon \right)]\;\;\\ \; & =E\left[\prod_{l=0}^{L-1}\mathrm{CN}\left(\bar{X}\left(l\right)|{\bar{A}}_{v}{\bar{S}}_{v}\left(l\right),\varepsilon^{-1}I_{2Q}\right)\right]+\ln p\left(\varepsilon \right)\\ \; & =LQ\ln\varepsilon -\varepsilon \sum_{l=0}^{L-1}E\left[∥\bar{X}\left(l\right)-{\bar{A}}_{v}{\bar{S}}_{v}{\left(l\right)∥}^{2}\right]+\ln p\left(\varepsilon \right)\\ \; & =LQ\ln\varepsilon -\varepsilon \sum_{l=0}^{L-1}{∥\bar{X}\left(l\right)-{\bar{A}}_{v}\mu_{l}∥}^{2}-\varepsilon \sum_{l=0}^{L-1}Tr\left({\bar{A}}_{v}^{T}{\bar{A}}_{v}\Sigma_{l}\right)-a\varepsilon . \end{array}$$

Let $\frac{\partial L(\varepsilon )}{\partial \varepsilon }=0$; ε will be obtained by

(50) $$\varepsilon =\frac{LQ}{a+\sum_{l=0}^{L-1}{∥\bar{X}\left(l\right)-{\bar{A}}_{v}\mu_{l}∥}^{2}+Tr\left({\bar{A}}_{v}^{T}{\bar{A}}_{v}\Sigma_{l}\right)}.$$

For δ and Λ, the following can be found:

(51) $$\begin{array}{cc} \; & L\left(\delta ,\Lambda \right)\propto E[\ln p\left({\bar{S}}_{v}|\delta ,\Lambda \right)p\left(\delta \right)p\left(\Lambda \right)]\\ \; & =E\left[\prod_{l=0}^{L-1}\mathrm{CN}\left({\bar{S}}_{v}\left(l\right)|0_{(P+2Q)\times 1},\Omega_{l}^{-1}\right)\right]+\ln p\left(\delta \right)+\ln p\left(\Lambda \right)\\ \; & =L\sum_{n=1}^{P}\ln\delta_{n}+\sum_{i=0}^{2Q-1}\sum_{l=0}^{L-1}\ln\lambda_{i,l}-\xi \sum_{n=1}^{P}\delta_{n}-\xi \sum_{i=0}^{2Q-1}\sum_{l=0}^{L-1}\lambda_{i,l}-\sum_{l=0}^{L-1}Tr\left(\left(\mu \mu_{l}^{T}+\Sigma_{l}\right)\Omega_{l}\right). \end{array}$$

Similarly, differentiate Equation (51) to $\delta_{n}$ and $\lambda_{i,l}$, respectively. They will be updated by:

(52) $$\delta_{n}=\frac{L}{\xi +\sum_{l=0}^{L-1}{\left[D_{l}\right]}_{n,n}},$$

(53) $$\lambda_{i,l}=\frac{1}{\xi +{\left[D_{l}\right]}_{i+P,i+P}},$$

where $D_{l}=Tr\left(\mu \mu_{l}^{T}+\Sigma_{l}\right)$.

For $\beta_{1}$, it can be updated through $\beta_{1}=V^{-1}\psi$ if V is invertible, given as:

(54) $$V=\left({\bar{B}}_{1}^{T}{\bar{B}}_{1}\right)⊙\left(\sum_{l=0}^{L-1}\left(\mu_{l}\mu_{l}^{T}+\Sigma_{l}\right)\right),$$

(55) $$\psi =\frac{1}{L}\sum_{l=0}^{L-1}\left(\mathrm{diag}\left(\mu_{l}\right)B_{1}^{T}\left(\bar{X}\left(l\right)-{\bar{A}}_{v}\mu_{l}\right)-\mathrm{diag}\left(B_{1}^{T}{\bar{A}}_{v}\Sigma_{l}\right)\right).$$

Additionally, if V is not invertible, $\beta_{1}$ can be updated by:

(56) $${\hat{\beta }}_{1}\left(n\right)=\frac{\psi \left(n\right)-V_{-n}^{T}\beta_{1,-n}}{V_{n,n}},$$

(57) $$\beta_{1}{\left(n\right)}^{new}=\left\{\begin{array}{cc} {\hat{\beta }}_{1}\left(n\right),\; & if\;{\hat{\beta }}_{1}\left(n\right)\in \left[-0.5r,0.5r\right]\\ -0.5r,\; & if\;{\hat{\beta }}_{1}\left(n\right)<-0.5r\\ 0.5r,\; & \mathrm{otherwise} \end{array}\right.,$$

where $\beta_{1}\left(n\right),\psi \left(n\right)$ are the *n*th elements of $\beta_{1}$, and ψ, respectively. $V_{-n}$ and $\beta_{1,-n}$ denote the operation which removes the *n*th entry of V and $\beta_{1}$.

Now, sparse reconstruction based SBL is realized by repeating the above procedure until it meets the terminal conditions. The update procedure is given in Algorithm 1. After sparse reconstruction, DOAs will be calculated based on the indexes of non-zero elements in $S_{out}$.
**Algorithm** **1** Proposed Method**Input:** 
Input signals X and sampling grid steering matrix $\bar{A}\left(\Theta \right)$ and its derivation $B_{v}$.**Output:** 
$S_{out}$1:Construct the virtual array signals $\tilde{X}$ and transform it to a real-valued model $\bar{X}$.2:Set the iteration number t=0 and initialize the value of different parameters as a=0.01, ξ=0.001, $\delta^{\left(0\right)}=I_{P\times 1}$, $\Lambda^{\left(0\right)}=I_{2Q\times L}$, $\beta_{1}^{\left(0\right)}=0_{(P+2Q)\times 1}$.3:Construct the off-grid steering matrix ${\bar{A}}_{v}=\bar{A}\left(\Theta \right)+B_{1}\mathrm{diag}\left(\beta_{1}^{\left(t\right)}\right)$.4:Let t=t+1 and calculate $\mu_{l}^{\left(t\right)}$ and $\Sigma_{l}^{\left(t\right)}$ for l=1,2,…,L by Equations (46) and (47).5:Calculate $\delta^{\left(t\right)}$ and $\Lambda^{\left(t\right)}$ by Equations (52) and (53).6:Calculate ε by Equation (50) and update $\beta_{1}^{\left(t\right)}$ by $\beta_{1}^{\left(t\right)}=V^{-1}\psi$ or Equation (57).7:Repeat step 3-6 until $∥\delta^{\left(t\right)}-\delta^{\left(t-1\right)}{∥}_{2}/{∥\delta^{\left(t-1\right)}∥}_{2}\leq 0.001$ or t>500.8:Output $S_{out}=\left[\mu_{1},\mu_{2},\ldots ,\mu_{L}\right]$.

## 4. Simulation Results and Analysis

In this section, a series of simulations are provided to evaluate the proposed method. A monostatic MIMO radar with seven transmitted arrays and six received arrays is considered. The simulation dataset has been partly attached in the Supplementary Materials. Moreover, in this dataset, two far-field narrow band BPSK signals are utilized as transmitted non-circular signals. DOAs of signals are considered as $\theta_{1}=45.4^{\circ }$ and $\theta_{2}=31.8^{\circ }$. The interval of the fixed sampling grid is set as $r=1^{\circ }$. Impulsive noise is induced by symmetric alpha-stable distribution. As the variance is not convergent for alpha-stable distribution, a generalized signal to noise ratio (GSNR) is usually utilized to measure the intensity of impulsive noise, given as

(58) $$\mathrm{GSNR}10\mathrm{lg}(P_{s}/\gamma ),$$

where $P_{s}$ is the signal power and γ is the dispersion parameter of alpha-stable distribution.

The comparison experiments are carried out in different parameters, mainly including characteristic exponent α, GSNR and signal snapshots. We compare our method with the three latest methods, mainly including nuclear norm minimization (NUC-NORM) [17], iterative reweighted proximal projection (IRPP) [18], robust variational Bayesian inference (RVSBL) [31] and multiple signal classification (MUSIC). NUC-NORM and IRPP are proposed for MIMO DOA estimation and RVSBL is proposed to deal with impulsive noise via Bayesian learning. Meanwhile, two quantities, named accuracy and root mean square error (RMSE), are calculated with 200 Monte-Carlo experiments. An accurate estimation of DOA is defined as:

(59) $$|{\hat{\theta }}_{k}\left(i\right)-\theta_{k}|\leq 2^{\circ },k=1,2,$$

where $\theta_{k}$ and ${\hat{\theta }}_{k}\left(i\right)$ denote the true DOA of the *k*th signal and the estimated DOA in the *i*th simulation, respectively. Then the accuracy can be obtained utilizing the ratio of the number of accurate estimations to the total number of experiments. RMSE will be achieved by:

(60) $$\mathrm{RMSE}=\sqrt{\frac{1}{N_{s}K}\sum_{i=1}^{N_{s}}\sum_{k=1}^{K}{\left({\hat{\theta }}_{k}\left(i\right)-\theta_{k}\right)}^{2}},$$

where $N_{s}$ is the number of accurate estimations for DOAs in the experiments.

### 4.1. Simulations versus GSNRs

**Figure 1:**
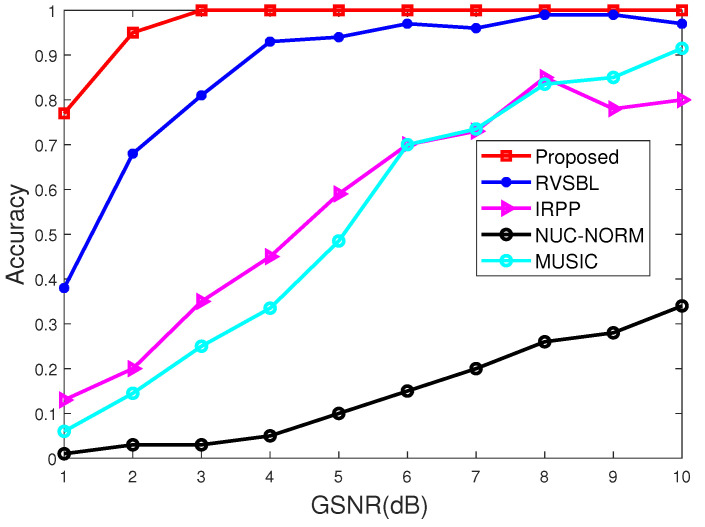
Accuracy with different GSNRs.

**Figure 2:**
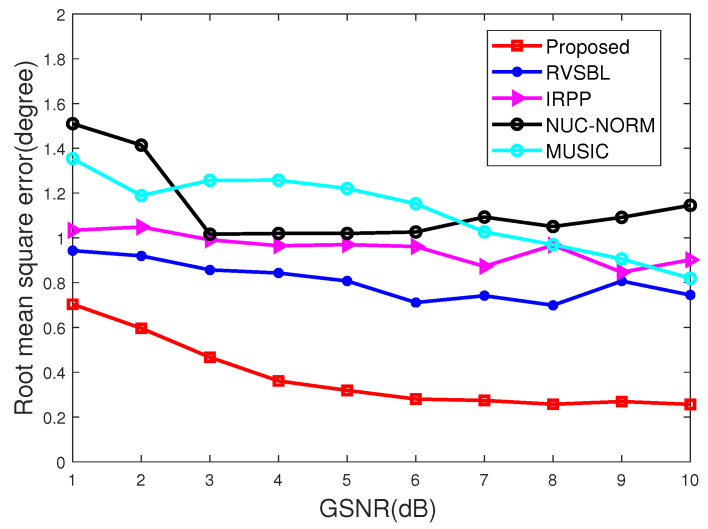
RMSE with different GSNRs.

In this subsection, we conduct the experiments with GSNR in a range from 1 dB to 10 dB. The characteristic exponent of noise is set as α=1.5, and the snapshot of signals is set as 10. Simulation results are illustrated in Figure 1 and Figure 2.

It is apparent from Figure 1 and Figure 2 that the proposed method outperforms the contrastive DOA methods under different GSNRs. The accuracy of the proposed method can converge to 1 when GSNR ≥ 3dB, while the contrastive methods require GSNR to be at least 8dB so as to achieve the same performance. Furthermore, RMSE for different methods is illustrated in Figure 2. It shows that our DOA method performs better than other contrastive methods. Among these methods, the proposed method can achieve the best performance in terms of both accuracy and RMSE since it can resist the influence of impulsive noise and off-grid effect. Thus, our method is more suitable for DOA estimation in different GSNRs.

### 4.2. Simulations versus Characteristic Exponents

**Figure 3:**
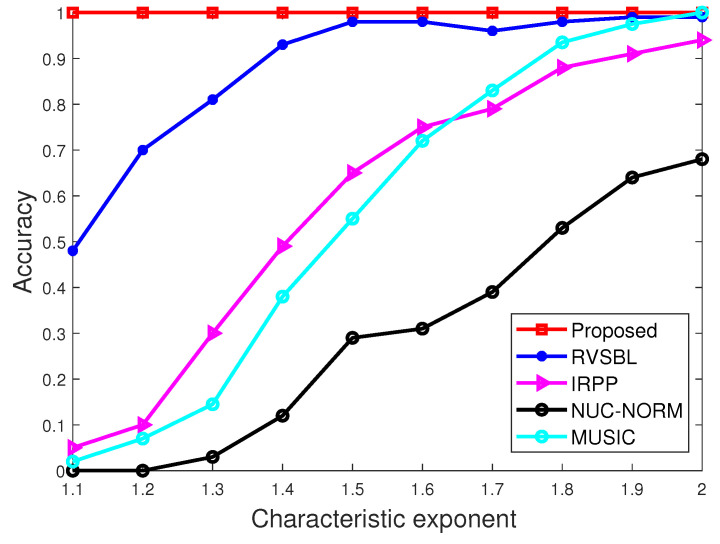
Accuracy with different characteristic exponents.

**Figure 4:**
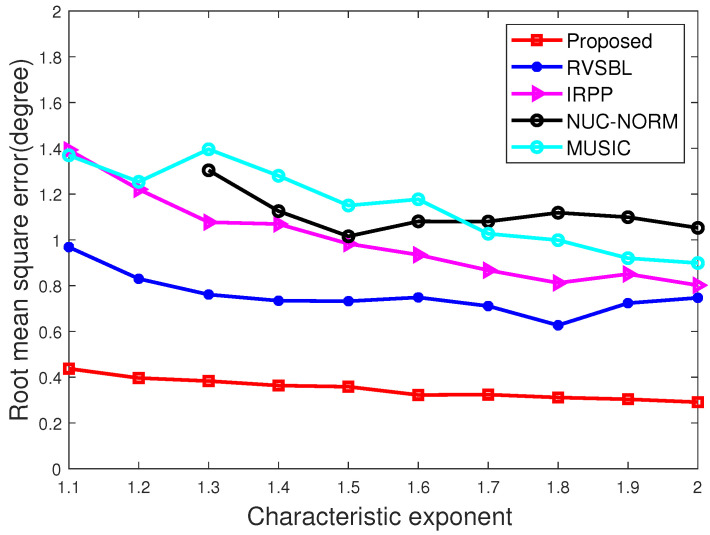
RMSE with different characteristic exponents.

In this subsection, we conduct the experiments with characteristic exponents α in a range from 1.1 to 2. GSNR remains unchanged at 5 dB. The snapshot of signals is set as 10. The results are demonstrated in Figure 3 and Figure 4.

As shown in Figure 3 and Figure 4, the performance of all methods become better when α increases. This is because the impulsiveness becomes weaker when α is larger. Clearly, compared with other contrastive methods, our method has higher accuracy in all characteristic exponent conditions. Moreover, we find only our method and RVSBL can keep good accuracies with small α since they have the resistance to impulsive noise. Meanwhile, our method shows a superior performance in terms of RMSE than other methods due to its effective consideration for the off-grid condition.

### 4.3. Simulations versus Signal Snapshot

**Figure 5:**
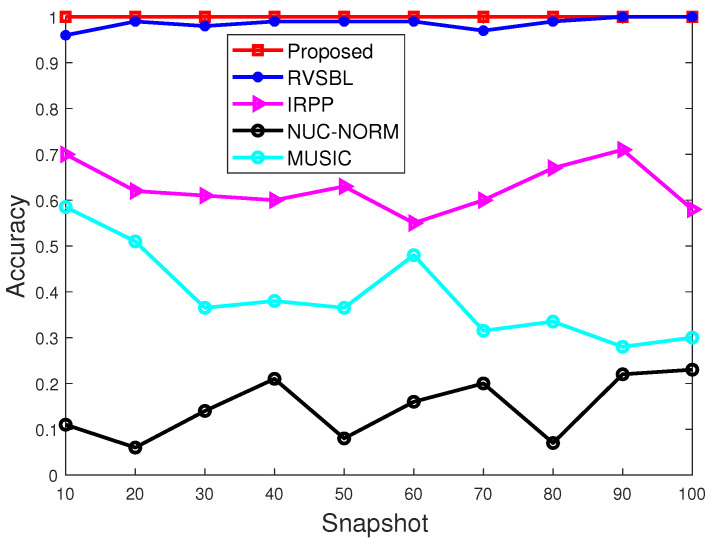
Accuracy with different snapshots.

**Figure 6:**
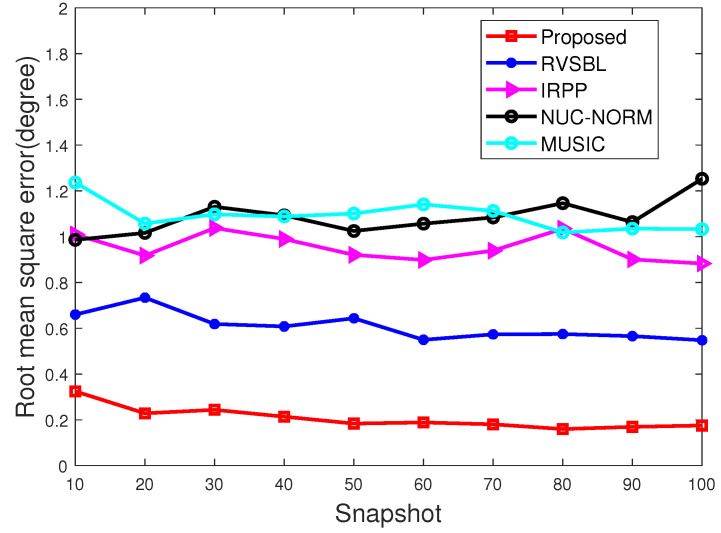
RMSE with different snapshots.

With this part, we make the performance comparison with different signal snapshots. Characteristic exponent and GSNR are set as α = 1.5 and 5 dB, respectively. The snapshot ranges from 10 to 100. Simulation results are given in Figure 5 and Figure 6.

As shown in Figure 5 and Figure 6, it can be observed that only our method and RVSBL can keep high accuracies in all conditions because of their suppression of impulsive noise. In addition, our method can even keep a totally successful estimation in all experiments. By contrast, IRPP- and NUC-NORM-based methods show bad and non-stable accuracy in the simulations. Furthermore, our method shows the best performance on RMSE due to its operation for off-grid condition.

### 4.4. Simulations versus DOAs

In this subsection, we mainly conduct the experiments with different DOAs of signals. Different DOAs and intervals are considered, and $\theta_{1},\theta_{2}$ are mainly set as $\left(31.8^{\circ },45.4^{\circ }\right)$, $\left(0.5^{\circ },8.7^{\circ }\right),\left(84.2^{\circ },73.5^{\circ }\right)$, $\left(0.8^{\circ },87.4^{\circ }\right)$. The characteristic exponents α is set as 1.5, and the snapshot of signals is set as 10. The results are demonstrated in Table 1.

It is apparent from Table 1 that the proposed method outperforms the contrastive DOA methods under different DOAs. The accuracy of the proposed method can achieve a superior performance under different DOAs and different intervals for a fixed samples grid, which is higher than the other contrastive methods. Among these DOA methods, the proposed method has the highest accuracy in terms of different intervals and DOAs even though DOAs are close to 0 and 90 degrees. Thus, our method has an outstanding performance in different DOAs.

Since the proposed DOA method and RVSBL show a better performance than other methods, the computation complexity of these two methods is compared. According to Ref. [31], the computation complexity of RVSBL is $O\left(P^{3}\right)$. The computation complexity of the proposed method is $O\left({\left(P+2Q\right)}^{3}\right)$. Since P>>Q, the complexities of two methods are comparable. Otherwise, with the help of real-valued transformation in the proposed method, the complexity can be reduced by a scale of four with the same matrix dimension. Therefore, the proposed method can show a better performance with proper computation complexity.

## 5. Conclusions

In this paper, an off-grid DOA estimation method for monostatic MIMO radar is proposed under impulsive noise. Based on the property of monostatic MIMO radar and non-circular signals, a real valued virtual array is constructed, which can extend the array aperture and reduce the computational complexity for Bayesian learning. Then, considering the property of impulsive noise, an off-grid sparse model is built for the virtual array. With the help of SBL, DOA estimation is realized by reconstructing the off-grid sparse model. A series of simulations is provided to verify the superiority of the proposed method, and simulation results indicate that the proposed method can lead to high accuracy and robust DOA estimation with monostatic MIMO radar in impulsive noise.

## Figures and Tables

**Table 1:** Accuracy with different DOAs of signals.

DOA Method	($31.8^{\circ },45.4^{\circ }$)	($0.5^{\circ },8.7^{\circ }$)	($84.2^{\circ },73.5^{\circ }$)	($0.8^{\circ },87.4^{\circ }$)
Proposed	100%	100%	98%	60%
RVSBL	93%	95%	88%	38%
IRPP	58%	50%	46%	3%
NUC-NORM	12%	10%	8%	2%

## Data Availability

The data presented in this study are partly available in the supplementary for the simulations.

## Supplementary Materials

The following supporting information can be downloaded at: https://www.mdpi.com/article/10.3390/s22166268/s1, Simulations versus GSNRs.
